# Evidence for a male‐biased sex ratio in the offspring of a large herbivore: The role of environmental conditions in the sex ratio variation

**DOI:** 10.1002/ece3.8938

**Published:** 2022-05-19

**Authors:** Robert Hagen, Sylvia Ortmann, Andreas Elliger, Janosch Arnold

**Affiliations:** ^1^ 28377 Leibniz Institute for Zoo and Wildlife Research Berlin Germany; ^2^ Wildlife Research Unit Agricultural Centre Baden‐Württemberg Aulendorf Germany

**Keywords:** *Capreolus capreolus*, effect size, Europe, fischer‘s principle, meta‐analysis, southern Germany

## Abstract

Numerous studies have examined whether the primary and/or secondary sex ratio in mammals, including humans, deviates from an equilibrium of 1:1. Although effect size in the sex ratio variation is expected to be low, a large sample size allows the identification of even small deviations from parity. In this study, we investigated whether the sex ratio of roe deer (*Capreolus capreolus*) offspring at birth approaches parity, using a large data set from roe deer offspring tagged in Baden‐Württemberg (Germany, 1972–2019, *N* = 12,437). In addition, a systematic re‐analysis of available data on the secondary sex ratios of roe deer was conducted to test whether our finding withstood the accumulation of further data. The null hypothesis that the sex ratio of roe deer (prenatal sex ratio and sex ratio at birth) approaches parity was rejected. Moreover, the secondary sex ratio of roe deer offspring deviated from the male‐biased mean for relatively cold or warm weather conditions during autumn and winter. Our study provides strong evidence for a male‐biased sex ratio in a large herbivore and weak evidence for variations in the secondary sex ratio owing to environmental conditions. The pattern is highly relevant in the context of climate change and its impact on the population dynamics of large herbivores.

## Graphical abstract

In this study, we showed that the secondary sex ratio of roe deer offspring is male biased and that the sex ratio deviated from the male‐biased mean during relatively cold or warm weather conditions during autumn and winter.

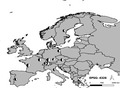

## 1 INTRODUCTION

The secondary sex ratio (sex ratio at birth) of males to females in large mammals is often interpreted as a dynamic equilibrium approaching a ratio of 1:1 (Fischer's principle—Fischer, 1930). This concept assumes that a temporally or spatially unbalanced sex ratio will be compensated at the population level through frequency‐dependent processes (Fischer, 1930). Even though an abrupt shift toward one sex is uncommon (West & Sheldon, 2002), skewed sex ratios have received considerable attention, which may bias the literature on sex ratios (Festa‐Bianchet, 1996; Palmer, 2000). Ecologists have proposed several hypotheses—relevant at the population and individual level, and neither incompatible nor mutually exclusive—regarding the production of a disproportionate number of male or female offspring (Clark, 1978; Gowaty & Lennartz, 1985; Hamilton, 1967; Leimar, 1996; Post et al., 1999; Trivers & Willard, 1973). However, in many cases, there is a disparity between the predicted and observed sex ratio (Cameron, 2004; Moore et al., 2005; Sheldon & West, 2004). A skewed sex ratio in large mammals has been attributed to active manipulation by the parents (references in Clutton‐Brock & Iason, 1986 or Hardy, 1997 or Frank, 1990, Douhard & Geffroy, 2021) or to stochastic errors (Hardy, 1997). Clutton‐Brock and Iason (1986, Page 340, first paragraph) suggested that variations in the sex ratio of non‐human mammals should be investigated based on at least 50 offspring.

In this study, we used data on both the prenatal sex ratio and the sex ratio at birth in European roe deer (*Capreolus capreolus*) to determine whether the sex ratio approaches parity. Unlike other large herbivores (Ripple et al., 2015), roe deer have not decreased in number throughout Europe since the early 1970s (Hagen et al., 2017). The species distribution range spans several different climatic zones, from southern Spain to northern Norway and from the Atlantic Ocean to the Black Sea (Linnell et al., 2020). Roe deer show little sexual size dimorphism and are only weakly polygynous (Vanpé et al., 2007). In roe deer, both sexes disperse (Debeffe et al., 2012; Pettorelli et al., 2003; Strandgaard, 1972). Female roe deer are mono‐estrous, i.e., they have only one estrous cycle per year (Linnell & Andersen, 1998 **)**. In central Europe, offspring are mainly born in May and June (Hagen et al., 2021; Plard et al., 2013) and the age of first parturition is typically 2 years (Hewison et al., 2005). After birth, male and female offspring receive equal care (Pelliccioni et al., 2004). Litter size in roe deer is between 0 and 3 (MacDonald & Johnson, 2008; van der Weijden & Ulbrich, 2020). A litter size of two is most common, with mixed litters (one male offspring and one female offspring) occurring more often than theoretically expected (MacDonald & Johnson, 2008). The authors found a male‐biased sex ratio in singletons (60% males, *N* = 416) and a sex ratio close to parity in twins (51% males, *N* = 2260) and triplets (50%, *N* = 66). Müri (1999) argued that weather conditions before the rut and during winter were linked to variations in the secondary sex ratio of roe deer offspring, as weather conditions affect the physiology of roe deer females (body weight), and that the mechanisms of roe deer reproduction influence the sex ratio at birth. In that study, the data of 3645 marked roe deer offspring tagged in Switzerland between 1971 and 1995 were analyzed and a negative correlation was found between the mean temperature in May and June (year *t *− 1) and the proportion of female offspring (year *t*) but a positive correlation between precipitation in January and February (year *t*) and the proportion of female offspring (year *t*). Thus, the assumption of Müri (1999) that weather conditions during early summer and during winter affect the sex ratio at birth is plausible based on the cascading effects of weather conditions (rut and winter) ‐> roe deer physiology ‐> sex ratio at birth. Several studies have shown that light mothers (i.e., very young and very old females) are less fertile (Gaillard et al., 1993; Hewison & Gaillard, 2001) and they produce an excess of male offspring (Focardi et al., 2002; Hewison et al., 1999; Hewison & Gaillard, 1996), whereas mothers in good physical condition have a higher fertility (Ellenberg, 1978; Focardi et al., 2002; MacDonald & Johnson, 2008) and produce more female offspring (Ellenberg, 1978; Focardi et al., 2002; Hewison et al., 1999). Hewison et al. (2005) reported that body mass is a good measure of phenotypic quality in roe deer because individuals have limited fat reserves (Toïgo et al., 2006). Thus, variation in the sex ratio at birth in roe deer will most likely depend on factors affecting either the body mass of very young (1‐year‐old) females and/or the proportion of female yearlings to all females.

In this study, we used the sex of 12,437 roe deer fawns marked in Baden‐Württemberg (southern Germany) from 1972 to 2019 to test the following hypotheses; (1) that the secondary sex ratio in roe deer is close to parity and (2) that variations in the secondary sex ratio are caused by environmental conditions (i.e., weather conditions). We expected a secondary sex ratio in roe deer of 1:1, according to Fischer's principle (Fischer, 1930), and that (i) the temperature in early summer (year *t *− 1) would negatively correlate with the proportion of female offspring (year *t*), and (ii) precipitation during winter (year *t*) would positively correlate with the proportion of female offspring (year *t*) (Müri, 1999). The robustness of the identified pattern based on a within‐population approach was then tested using a between‐population approach based on data obtained from a systematic literature review (Vetter et al., 2013). For the latter, information on both the prenatal sex ratio (sex ratio before birth) and the secondary sex ratio were used to test whether these ratios were close to parity and whether variations in the sex ratio were associated with the longitude and latitude of the particular study site.

## 2 MATERIALS AND METHODS

### 2.1 Baden‐Württemberg (Germany)

Data on the secondary sex ratio in roe deer originated from a long‐term project that has been continuously conducted in Baden‐Württemberg (federal state in southern Germany) since 1970 (Hagen et al., 2021). Individuals are marked according to national guidelines for the care and use of animals (Jagd‐ und Wildtiermanagementgesetz (JWMG)–federal law of Baden‐Württemberg). The sex of roe deer offspring after birth was reported in Baden‐Württemberg (Germany) by volunteers and hunters. For 3280 of 12,437 marked fawns, the sex was evaluated when the tagged individual died in subsequent years. For <3% of 3280 marked individuals, the determined sex had to be corrected at death (65 individuals were male but had been marked as female, and 32 individuals were female but had been marked as male). The mean estimated age of these 12,437 roe deer offspring was 8.43 days (median = 8 days, standard deviation = 2.5 days).

We then tested whether the sex ratio variation in roe deer in Baden‐Württemberg was associated with weather conditions during the early summer and winter, as reflected by monthly mean values of temperature (*T*) and precipitation (*P*) (Müri, 1999). A generalized linear model (binomial) was established using sex (0 – male, 1 – female) as the response variable and the monthly mean values of temperature and precipitation as predictor variables (Equation 1):

(1) $$\text{sex}∼T_{\text{Jan}(t)}+T_{\text{Feb}(t)}+T_{\text{March}(t)}+T_{\text{May}(t‐1)}+T_{\text{June}(t‐1)}+T_{\text{Dec}(t‐1)}+P_{\text{Jan}(t)}+P_{\text{Feb}(t)}+P_{\text{March}(t)}+P_{\text{May}(t‐1)}+P_{\text{June}(t‐1)}+P_{\text{Dec}(t‐1)}$$

While predictor variables were suggested by Müri (1999), we further used a systematic, more holistic approach to identify the strongest periods of climatic sensitivity for sex ratio variation, by using the monthly mean values of temperature and precipitation between June (*t *− 1) and February (*t*) (Bailey & van de Pol, 2016). The analysis was conducted for four different height classes (<250 m, 250–500 m, 500–750 m, and ≥750 m, Hagen et al. (2021)) as we assumed that the climatic sensitivity of roe deer individuals living under different environmental conditions is not the same. The influence of weather variables on the offspring sex ratio of roe deer was analyzed using the R‐package “climwin” considering quadratic polygons and a generalized linear model (binomial) as the null model (Bailey & van de Pol, 2016). The model weights across all tested periods and all height classes were compared.

On the basis of the results obtained in the climwin analysis, the following generalized linear model (binomial) was used:

(2) $$\begin{array}{c} \begin{array}{c} \text{sex}∼f\left(T_{\text{Climate window}}\right)+f\left(P_{\text{Climate window}}\right)\\ |\text{with}\;f\left(x\right)=a_{0}+a_{1}x+a_{2}x^{2}\;\text{and sex as the response variable}\;\left(0-\text{male},\;1-\text{female}\right). \end{array} \end{array}$$

Since the data on the average monthly temperature in Baden‐Württemberg do not take into account the height above sea level (*hasl)*, a spatially explicit variable, the standard environmental lapse rate (Thayyen & Dimri, 2018), was calculated (Equation 3) and applied in Equation 2:

(3) $$\begin{array}{c} T_{\mathrm{cor}}\left[{}^{\circ}C\right]=T\left[{}^{\circ}\;C\right]‐0.65\left[{}^{\circ}C\right]*hasl\left[m\right]/100\left[m\right] \end{array}$$

Data on *hasl* were obtained from the “Landesamt für Geoinformation und Landentwicklung Baden‐Württemberg” (AZ 2851 9‐1/3) and covered a grid size of 250 × 250 m. The generalized linear models were run using R version 3.6.0 (R Core Team, 2019) and the R‐package “lme4” (Bates et al., 2015). A *p* < .05 was defined as statistically significant.

### 2.2 Systematic literature review

Data on the sex ratio in roe deer originated from a systematic literature review. The literature search was based on a breadth first search conducted in July 2021 using the “Web of Science” and “Google Scholar”. Keywords included “roe deer” or “capreolus capreolus” and “sex ratio at birth” or “primary sex ratio” or “secondary sex ratio” (Web of Science) and “sex ratio” and “roe deer” (Google Scholar). The results were scanned and the findings checked for their relevance for our database based on whether the article contained the words “sex ratio” and “roe deer” in the abstract and provided data on the primary (prenatal sex ratio) or secondary sex ratio of roe deer (for Google Scholar the first 100 results were scanned). In addition, the title of each article cited was checked to determine whether it contained at least one of the key words “roe deer” or “sex ratio.” For each data set, the following information was collected from the reference: sample size (roe deer offspring), country, specific location and its longitude and latitude, whether the roe deer lived in enclosures, in natural habitats, or under island conditions (islands, fenced area, or isolated populations), and whether the prenatal sex ratio or secondary sex ratio was documented. Data sets that included data on the sex ratio of roe deer offspring older than 6 weeks were excluded, as those ratios might also have reflected sex‐specific differences in offspring mortality, such as due to predation. However, even though sex‐specific mortality may occur directly after birth, 6 weeks was set as the cut‐off value, as the age of the oldest marked individual in Baden‐Württemberg was estimated to be 42 days (6 weeks). Data sets and/or references with a sample size of <50 offspring were also excluded (Clutton‐Brock & Iason, 1986, Page 340, first paragraph). If the data on the sex ratio at birth at one location had been published in several publications, the more holistic data collection or a combination of these data sets was used. For both the data sets and references that included data on the prenatal or secondary sex ratio, both the odds ratio (Mengersen & Gurevitch, 2013), determined from the proportion of female offspring (*pf*), and its 95% confidence interval were calculated using the exact binomial test. The effect size *d* was calculated as (Chinn, 2000):

(4) $$\begin{array}{c} d=LN\left(\mathrm{OR}\right)/\left(\pi /\surd 3\right)\\ |\text{with}\;OR=\left(pf/\left(1‐pf\right)\right) \end{array}$$

Thus, a value of *d* < 0 reflected a male‐biased sex ratio. Random and fixed models were then used to estimate the overall effect size. A counter‐enhanced funnel plot was generated to visualize the effect size and its standard error (Peters et al., 2008). The quantity *I* ^2^ was calculated to quantify the heterogeneity among data sets and publications (Higgins et al., 2003). Tests were conducted for an association of the effect size (*d*) with the longitude and latitude of each study site and thus for spatial differences in the weather conditions throughout Europe. The generalized linear models were run using R version 3.6.0 (R Core Team, 2019) and the R‐package “lme4” (Bates et al., 2015). A *p* < .05 was defined as statistically significant. The meta‐analysis was conducted using the R‐package “metafor” (Viechtbauer, 2010).

## 3 RESULTS

### 3.1 Baden‐Württemberg (Germany)

The generalized linear model (Equation 1) using the same set of parameters as in Müri (1999) revealed that none of those parameters (monthly mean precipitation in May, June, January, and February and monthly mean temperature for May and June) was associated significantly with the proportion of female offspring in Baden‐Württemberg (Appendix Table S3). The application of “climwin” suggested that sex ratio of roe deer (*t*) is affected by precipitation in September (*t *− 1) (Appendix Figure S7) and by specific time periods for temperature according to height above sea level (Appendix Figure S8). However, a search for a time period that covered the relevant periods for all height classes revealed that the temperature between October (*t *− 1) and February (*t*) was the most likely to induce variations in roe deer sex ratio (Appendix Figure S8).

**FIGURE 1:**
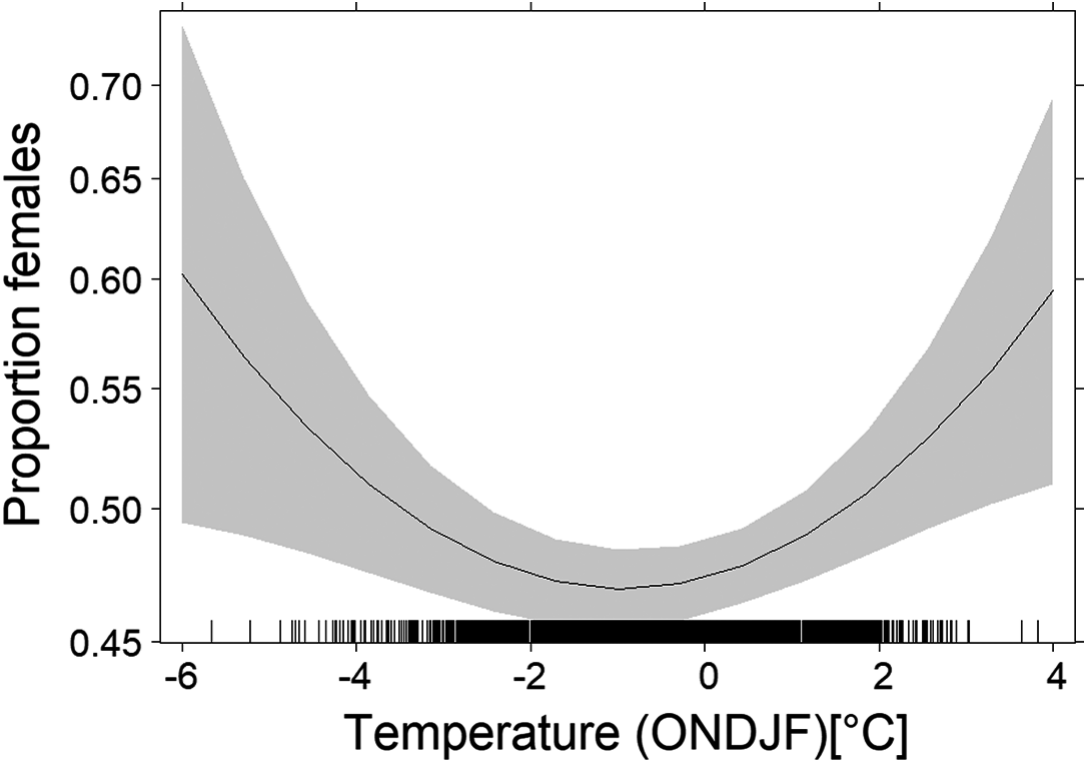
Effect plot of the proportion of female offspring and temperature (October, November, December, January, February) for Baden‐Württemberg (Germany). The gray‐shaded area represents the 95% confidence interval (number of tagged offspring equals 12,437)

Specifically, either relatively cold or relatively warm weather conditions seemed to favor conditions that induce deviations from the male‐biased mean value; by contrast, the variable precipitation in September was not significant (Table 1; Figure 1). However, because the secondary sex ratio of marked roe deer offspring might be impacted by the mortality of roe deer offspring within the first weeks after birth, before they would have been marked, the results shown in Figures S5 and S6 cover all roe deer offspring <3 days and all roe deer offspring <7 days.

**TABLE 1:** Results of Equation 2 using the sex of 12,437 roe deer offspring as the response variable based on parameter estimates to three decimal places and the standard error of those estimates. The relationship between the proportion of female offspring and the mean temperature (Oct, Nov, Dec, Jan, and Feb) is depicted in Figure 1

Variable	Parameter estimate	Standard error	*p*‐value
Intercept	−0.855	0.068	
Precipitation (Sep)	0.003	0.002	.11
Precipitation (Sep)^2^	0	0	.13
Temperature (Oct–Feb)	0.019	0.084	.03
Temperature (Oct–Feb)^2^	0.001	0	.01

### 3.2 Systematic literature review

In addition to the two unpublished data sets for roe deer offspring in Baden‐Württemberg and Niederfinow (Germany), data were obtained in a systematic literature search, which yielded 46 references (89 data sets) with information on the sex of roe deer offspring (Appendix Table S1). Of those, 14 references were obtained directly via our literature search, 29 from reference lists of other authors, and 3 references from the authors. For one reference (reference 3, Appendix Table S1), we had no direct access and one reference did not provide precise information on whether the prenatal or the secondary sex ratio was determined (reference 26, Appendix Table S1). For eight references (reference 13, 24, 27, 29, 31, 33, 38, and 43, Appendix Table S1), the data were published in a more holistic data collection. In two cases, data sets of two references were combined (reference 35 and 37; 32 and 39, Appendix Table S1 **)**. At the level of data sets, 13 data sets were excluded because the roe deer offspring were older than 6 weeks (data sets 1, 4, 10, 16, 22–25, 32, 30–31, 66, and 89, Appendix Table S1). Additionally, 14 data sets (data sets 7, 13, 18–19, 21, 34–35, 38, 40, 44, 52, 65, 71, and 88, Appendix Table S1, the bold numbers correspond to references 7, 14, 28, and 45) and thus 4 references were excluded because the sample size in the respective study was <50 offspring (Clutton‐Brock & Iason, 1986). For the remaining 51 data sets (25 references), the proportion of female offspring (Table 2) and the effect size were calculated.

**TABLE 2:** Proportion of female offspring for each reference (calculated using data with two decimal places), its 95% confidence interval, and information on the sample size, country, living conditions, author, and year of publication

Reference	Proportion of female offspring	CI_95_	Sample Size	Country	Living conditions (Free ranging—F, island condition—FI, and enclosures—E)	Prenatal (P) or secondary (S) sex ratio	Author and Year of publication
2	0.48	[0.41,0.55]	203	Switzerland	F	S	Kurt and Sägesser, 1966
5	0.44	[0.4,0.48]	679	Switzerland	F	S	Kurt, 1968
6	0.47	[0.34, 0.61]	55	Great Britain	F	P	Prior, 1968
8	0.56	[0.46, 0.65]	108	Sweden	F	S	Borg, 1971
8	0.47	[0.43, 0.52]	475	Sweden	F	P	Borg, 1971
9	0.47	[0.41, 0.53]	265	Denmark	F	S	Strangaard, 1972
10	0.57	[0.48, 0.66]	117	Germany	F	P	Georgii, 1973
11	0.45	[0.36, 0.49]	262	Switzerland	F	P	Wandeler, 1975
15	0.47	[0.39, 0.54]	193	Germany	E	S	Ellenberg, 1978
16	0.56	[0.46, 0.65]	111	Poland	F	P	Fruzinski and Labudski, 1982
17	0.46	[0.4, 0.52]	298	Poland	F	P	Kaluziński, 1982
18	0.48	[0.4, 0.55]	116	Germany	F	P	Stubbe et al., 1982
19	0.5	[0.46, 0.54]	763	Austria	F	S	Engl, 1982
23	0.48	[0.45, 0.5]	1600	Great Britain	F	P	Hewison, 1993
24	0.49	[0.41, 0.58]	142	Belgium	F	P	Wauters et al., 1995
30	0.51	[0.45, 0.57]	293	Norway	FI	S	Linnell & Andersen, 1998
34	0.55	[0.46, 0.64]	134	Spain	F	S	Quesada and Carranza, 2000
35,37	0.55	[0.46, 0.63]	132	Italy	F	S	Focardi et al., 2002; Pelliccioni et al., 2004
36	0.48	[0.45, 0.51]	1235	France	FI	S	Pettorelli et al., 2003
32,39	0.47	[0.46, 0.49]	5315	Switzerland	F	S	Müri, 1999; Signer and Jenny, 2006
40	0.6	[0.48, 0.72]	73	Hungary	F	P	Majzinger 2006
42	0.47	[0.46, 0.49]	2613	Great Britain	F	P	MacDonald and Johnsen 2008
44	0.49	[0.46, 0.52]	1083	France	FI	S	Plard et al., 2014
47	0.48	[0.47, 0.49]	12473	Germany	F	S	Hagen et al., this publication
48	0.51	[0.44, 0.57]	223	Germany	E	S	Ortmann, personal communication

**FIGURE 2:**
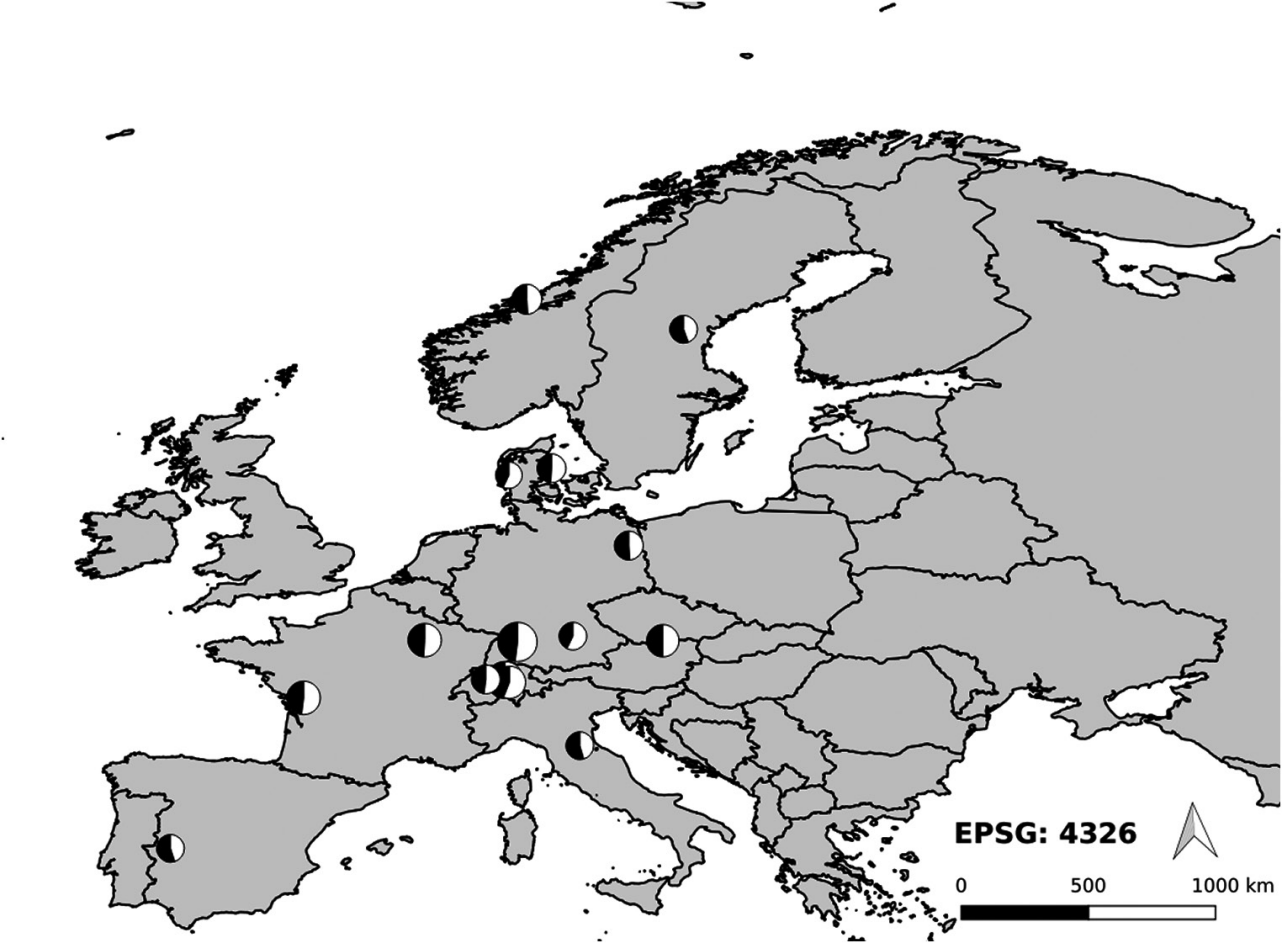
Locations where the secondary sex ratio of roe deer offspring was documented (cf. Table 3 and Figure 4). The size of each pie chart is exponentially proportional to the number of offspring. The proportion of male offspring is represented by the white fraction of each pie

For data sets with information on the prenatal sex ratio (Appendix Table S2), the distribution by country was (36 data sets): Great Britain (28), Germany and Poland (2 each), and Belgium, Hungary, Sweden, Switzerland (1 each). For data sets with information on the secondary sex ratio (Table 3), the distribution by country was (15 data sets): Germany and Switzerland (3 each), Denmark and France (2 each), and Austria, Italy, Norway, Spain, and Sweden (1 each) (Figure 2).

**TABLE 3:** Proportion of female offspring for each study site with information on the secondary sex ratio of roe deer offspring (calculated using data with two decimal places), its 95% confidence interval, and information on the sample size, location, coordinates (in case of several distinct locations, a representative coordinate was chosen), and living conditions. Bold entries refer to deviations from parity

ID (see Table S1 for further information)	Reference	Proportion of female offspring	CI_95_	Sample Size	Location	Coordinate in WGS 84 (Lat, Long)
2	2	0.48	[0.41,0.5]	203	Diverse (Switzerland)	46.95, 7.62
**5**	**5**	**0.44**	**[0.4, 0.48]**	**679**	**Diverse (**Switzerland**)**	**46.82, 8.63**
9	8	0.47	[0.43, 0.52]	475	Diverse (Sweden)	62.39, 16.32
11	9	0.49	[0.41, 0.57]	168	Kalø (Denmark)	56.29, 10.48
12	9	0.43	[0.33,0.54]	97	Borris (Denmark)	55.95, 8.66
20	15	0.43	[0.35, 0.51]	147	Stammham (Germany)	48.86, 11.46
29	19	0.5	[0.46, 0.54]	763	Diverse (Austria)	48.65, 15.39
52	28	0.51	[0.45, 0.57]	293	Storfosna (Norway)	63.67, 9.41
58	34	0.55	[0.46, 0.64]	134	Caceres (Spain)	39.51, −6.26
59 and 61	35,37	0.55	[0.46, 0.63]	132	Tredozio (Italy)	44.05, 11.74
60	36	0.48	[0.45, 0.51]	1235	Chizé (France)	46.13, −0.4
**56 and 63**	**32,39**	**0.47**	**[0.46, 0.49]**	**5315**	**Diverse** (Switzerland)	**46.93, 8.45**
87	44	0.49	[0.46, 0.52]	1083	Trois Fontaine (France)	48.70, 4.93
**90**	**47**	**0.48**	**[0.47, 0.49]**	**12,473**	**Diverse** (Germany)	**48.6, 9.01**
91	48	0.51	[0.44, 0.57]	223	Niederfinow (Germany)	52.85, 13.91
In total				23,053		

**FIGURE 3:**
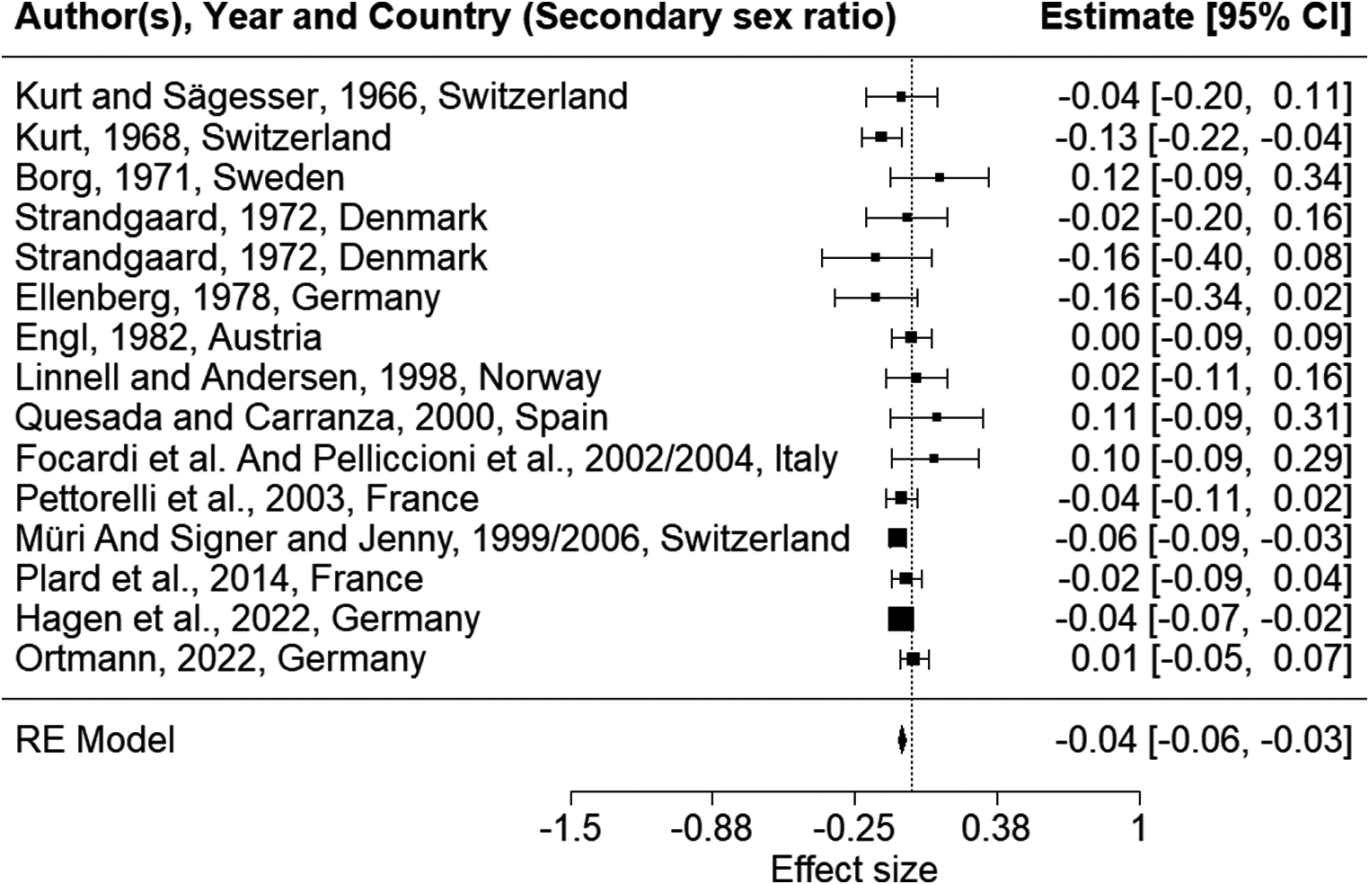
Calculated effect sizes for the proportion of female offspring among roe deer offspring at birth and its confidence interval (cf. Tables 1 and 2). The size of the square is proportional to the logarithm of the number of offspring. Values < 0 (vertical dashed line) reflect a male‐biased sex ratio

We used a random‐effect model to calculate the overall estimates for the effect size for data sets as −0.06 (CI_95_ [−0.09, −0.02]) for the prenatal sex ratio and −0.04 (CI_95_ [−0.06, −0.03] for the secondary sex ratio (Figure 3 and Appendix Figure S1), and a fixed model to calculate the overall estimates for the effect size for references as −0.05 (CI_95_ [−0.07, −0.02]) for the prenatal sex ratio and −0.04 (CI_95_ [−0.06, −0.03] for the secondary sex ratio. Although the magnitude of the overall effect was small, the calculated confidence intervals indicated a male bias in the sex ratio of roe deer offspring.

**FIGURE 4:**
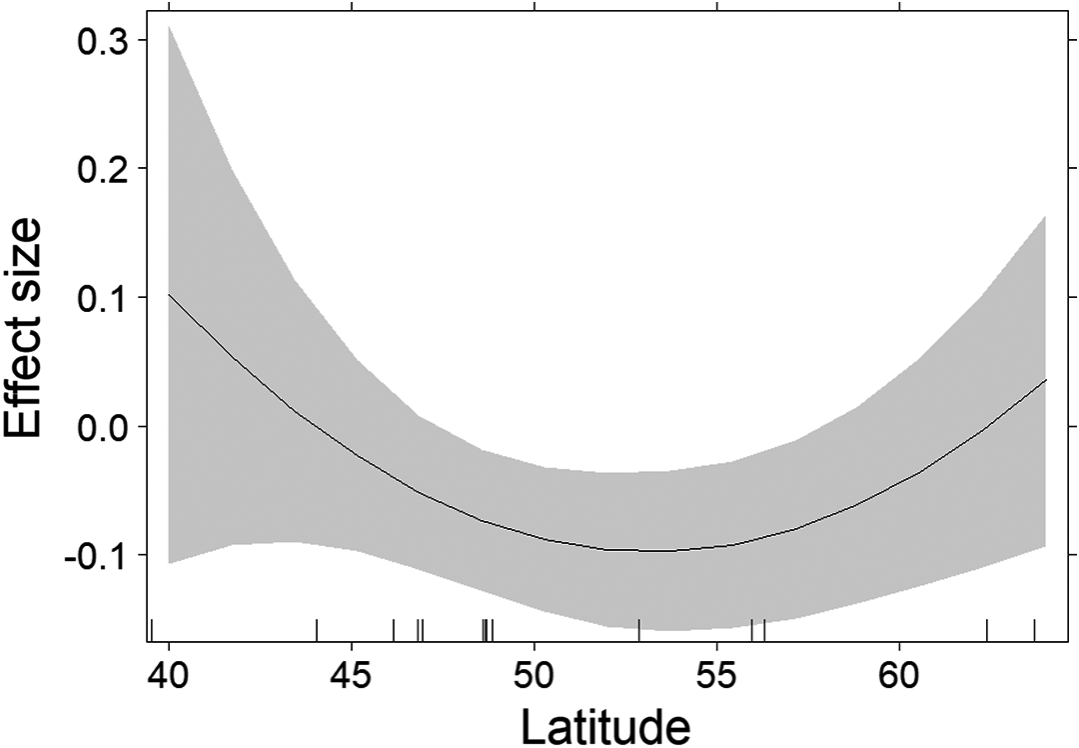
The calculated effect size for references containing data on the secondary sex ratio vs. latitude (based on 15 data sets, 23,053 roe deer offspring; cf. Figure 3). A negative effect size reflects a male‐biased sex‐ratio

The calculated effect sizes did not differ between the prenatal and secondary sex ratios. The funnel plots revealed a symmetric distribution (Appendix Figure S2) and the quantity *I* ^2^ was 0.16% (data sets on the secondary sex ratio) and 11.4% (data sets on the prenatal sex ratio). These results indicated no obvious publication bias and a low heterogeneity among data sets (Higgins et al., 2003). For the secondary sex ratio, a positive effect size and thus a female‐biased sex ratio coincided with either low or high latitudes (Figure 4; Table 4; Appendix Figure S3), although this association was not significant. Moreover, the effect size for the secondary sex ratio was not associated with longitude (Table 4; Appendix Figure S4).

**TABLE 4:** Relationship between the effect size (*d*) and the latitude based on parameter estimates to three decimal places and the standard error of those estimates

Variable	Parameter estimate	Standard error	*p*‐value
Intercept	3.114	1.503	—
Latitude	−0.121	0.057	.06
Latitude^2^	0.001	0.001	.06
Longitude	−0.002	0.008	.78
Longitude^2^	0.001	0.001	.22

## 4 DISCUSSION

The results of this study suggest that the secondary sex ratio in a large European herbivore, roe deer, is male biased and that deviations from this bias are sensitive to variations in the weather conditions during autumn and winter. In numerous species, the secondary sex ratio (male:female) deviates from 1:1 (Butka & Freedberg, 2019; Gowaty & Lennartz, 1985; Robert & Schwanz, 2011) but there is little evidence that this deviation persists for a given species when large data sets are analyzed (Palmer, 2000) or when the variation in the secondary sex ratio of free‐ranging mammals is linked to environmental factors (but see Kruuk et al., 1999). However, our finding of a male‐biased sex ratio in roe deer was robust to the addition of data on the sex ratio of roe deer offspring (Table 2; Figure 2), which led us to investigate conditions for which the sex ratio in roe deer deviates from this expectation. An analysis of spatially explicit information for each offspring tagged in Baden‐Württemberg (Germany) showed that comparatively cold or comparatively warm weather conditions during autumn and winter (October–February) were associated with a female‐biased sex ratio in roe deer (Table 1; Figure 1). Moreover, the effect size calculated in our meta‐analysis of the secondary sex ratio was weakly associated with the latitude of the study site (Table 4). A female‐biased sex ratio was documented for Southern and Northern Europe and thus, for regions characterized either by mild or by severe weather conditions during autumn and winter (Figure 4). Thus, even if not statistically significant, the available information on the secondary sex ratio in roe deer points to a role for environmental conditions in the sex ratio variation at birth (Figures 1 and 4).

Previous studies found that the sex ratio of roe deer offspring depends on the body weight of the female, with light females tending to give birth to male singletons (Focardi et al., 2002; Hewison et al., 2005), and females in better condition to twins or triplets with comparable fractions of male and female offspring (Flajšman et al., 2017; MacDonald & Johnson, 2008). Similar results have been reported for white‐tailed deer in North America (McGinley, 1984). The proportion of light females was shown to largely correspond to the proportion of young females, which on average have a lower body weight (Mysterud & Østbye, 2006). It has also been shown that among reproductive females, the proportion of 2‐year‐olds is higher than that of 8‐year‐old females (40% vs. 4% Andersen, 1953). Temperature affects the body mass of ungulates in Europe (Herfindal et al., 2020) and that of roe deer during the winter period (Kałuziński, 1982; Mysterud & Østbye, 2006; Stubbe, 1963). Hewison and Gaillard (2001) found a significant influence of the temperature in winter on litter size in two of nine roe deer populations. In this context, our findings indicate that a continuation of the recent rather mild weather conditions in most of Europe, attributed to climate change, will initiate a trend toward a female‐biased sex ratio for roe deer populations throughout Europe. The physiological mechanism responsible for sex ratio variation in roe deer and in large mammals, in general, remains poorly understood (Merkling et al., 2018 but see Edwards et al., 2016; Douhard, 2017). Our results on the role of weather conditions in inducing a variation in the sex ratio suggest that the physical condition of females before (October, November), during (December, January), and after embryo implantation (February) is a major driver of the sex ratio variations in roe deer. The non‐linear pattern between the secondary sex ratio and temperature identified in this study might be related to sex‐based differences in the mortality of newborns in Southern and Northern Europe. This assumption is based on the differences in the prenatal and secondary sex ratios of roe deer (Appendix Figure S3). The plot of the effect size for the prenatal sex ratio vs. latitude was consistent with a linear and negative association between the two variables.

Our results on the male‐biased sex ratio in roe deer and deviations of this expectation in the years following severe or mild weather conditions during autumn and winter can help to explain why weather conditions act as a density‐independent force contributing to the persistence of spatial gradients and to synchronized temporal variations in roe deer bag records (Hagen et al., 2017). Broader confirmation of our results regarding the impact of weather on the sex ratio of roe deer offspring would point to an increase in the reproductive potential of roe deer throughout their distribution range during recent decades.

## AUTHOR CONTRIBUTION

**Robert Hagen:** Conceptualization (lead); Data curation (equal); Formal analysis (lead); Investigation (lead); Methodology (lead); Writing – original draft (lead); Writing – review & editing (equal). **Sylvia Ortmann:** Data curation (supporting); Methodology (supporting); Writing – review & editing (equal). **Andreas Elliger:** Data curation (supporting); Project administration (equal); Writing – review & editing (supporting). **Janosch Arnold:** Conceptualization (supporting); Project administration (equal); Supervision (lead); Writing – review & editing (supporting).

## Supporting information

Fig S1

Fig S2

Fig S3

Fig S4

Fig S5

Fig S6

Fig S7

Fig S8

Table S1‐S3

Table S4

## Data Availability

Data are archived in the Zenodo repository, https://doi.org/10.5281/zenodo.6513932.
