# Corrigendum

**DOI:** 10.1002/ece3.9595

**Published:** 2022-12-13

In the recent article by Hagen et al. (2022), the authors would like to add who funded the publication of the article in the Acknowledgments section.

It should have read as follows:

## References

[ece39595-bib-0001] Hagen, R. , Ortmann, S. , Elliger, A. , & Arnold, J. (2022). Evidence for a male‐biased sex ratio in the offspring of a large herbivore: The role of environmental conditions in the sex ratio variation. Ecology and Evolution, 12, e8938. 10.1002/ece3.8938 35600697 PMC9120210

