# Supplementary figures and images for: Evidence for a male‐biased sex ratio in the offspring of a large herbivore: The role of environmental conditions in the sex ratio variation
Source: Ecol Evol. 2022 May 19;12(5):e8938. doi: 10.1002/ece3.8938 (PMC9120210; doi:10.1002/ece3.8938)

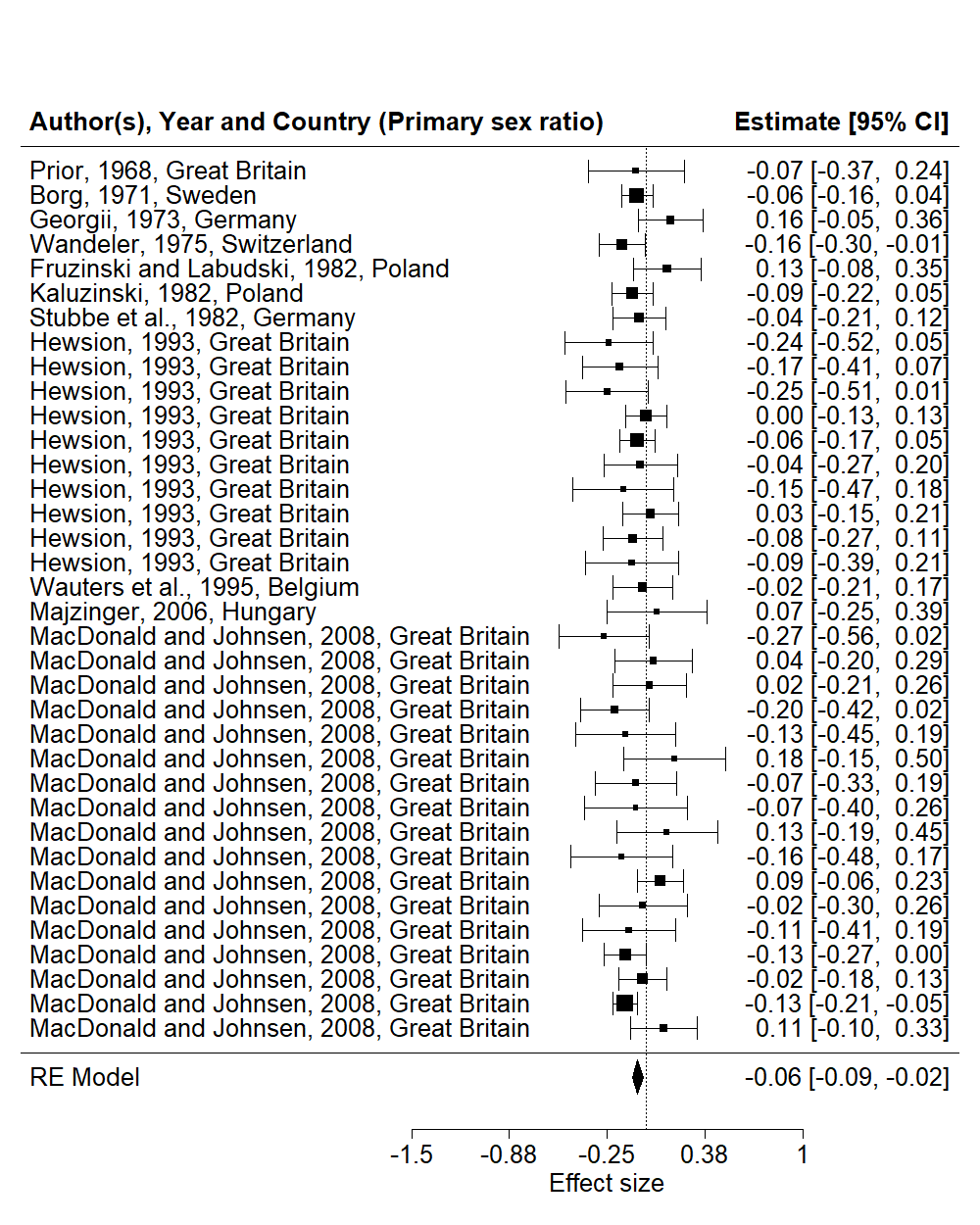

Supplement: Supplementary file 1 — Fig S1 [file ECE3-12-e8938-s010.tiff]

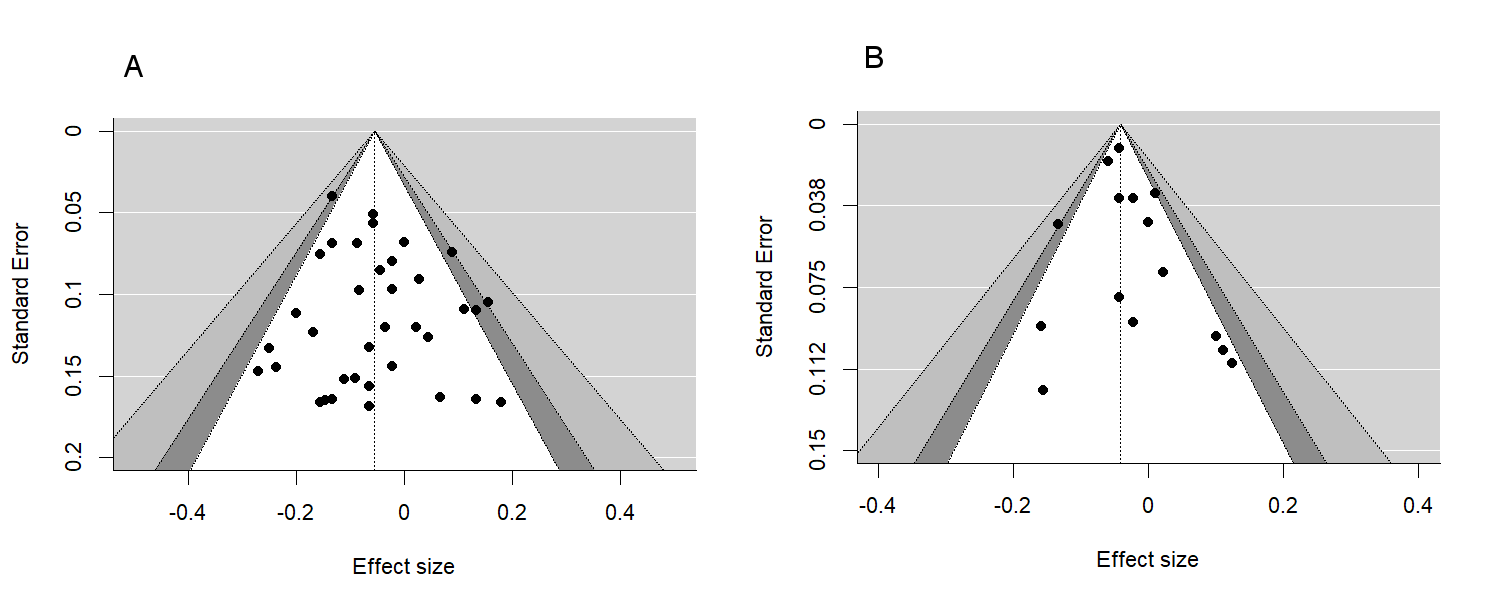

Supplement: Supplementary file 2 — Fig S2 [file ECE3-12-e8938-s004.tif]

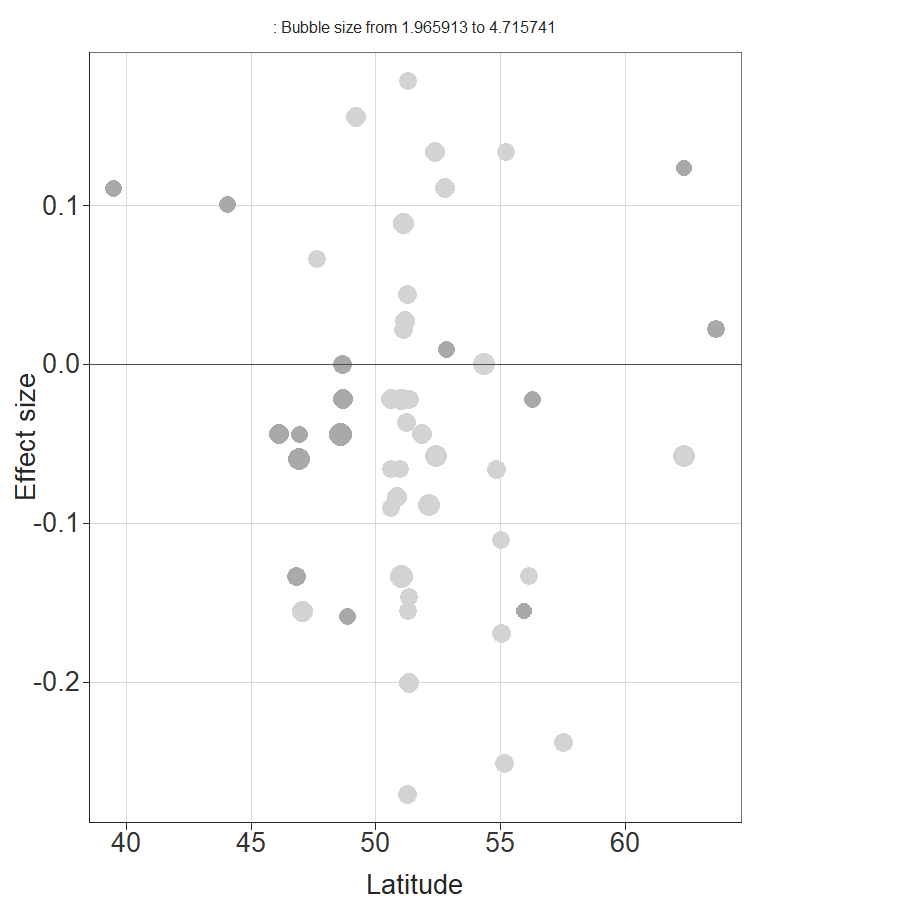

Supplement: Supplementary file 3 — Fig S3 [file ECE3-12-e8938-s007.tiff]

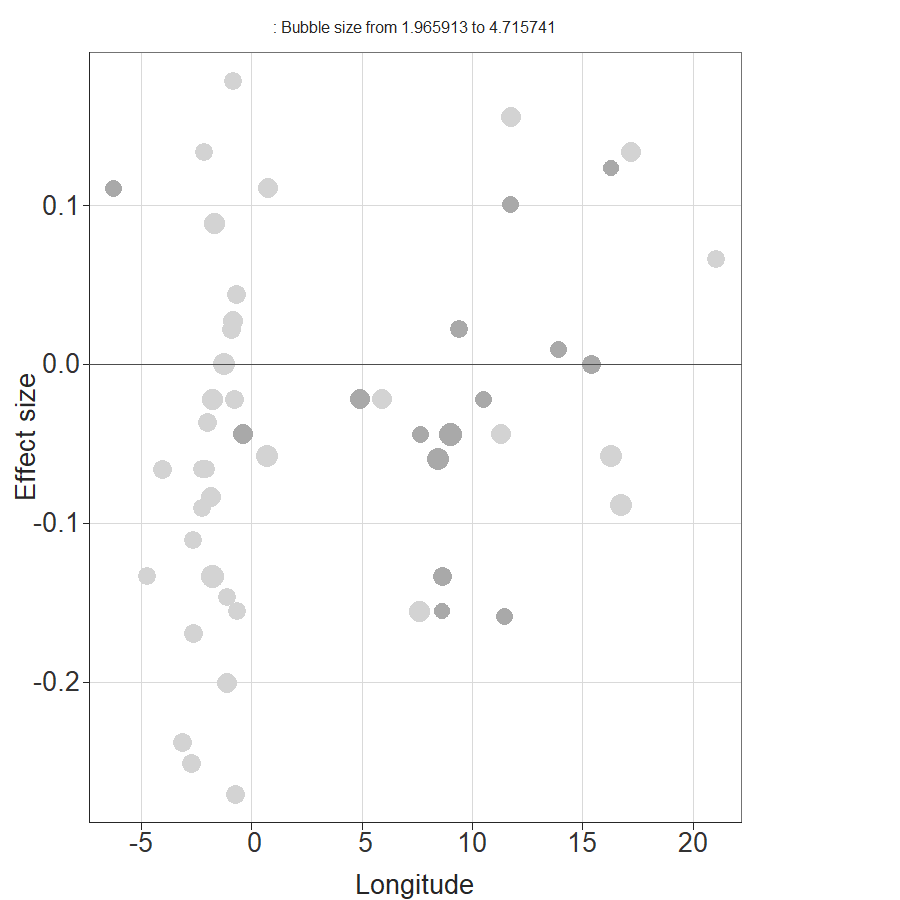

Supplement: Supplementary file 4 — Fig S4 [file ECE3-12-e8938-s003.tiff]

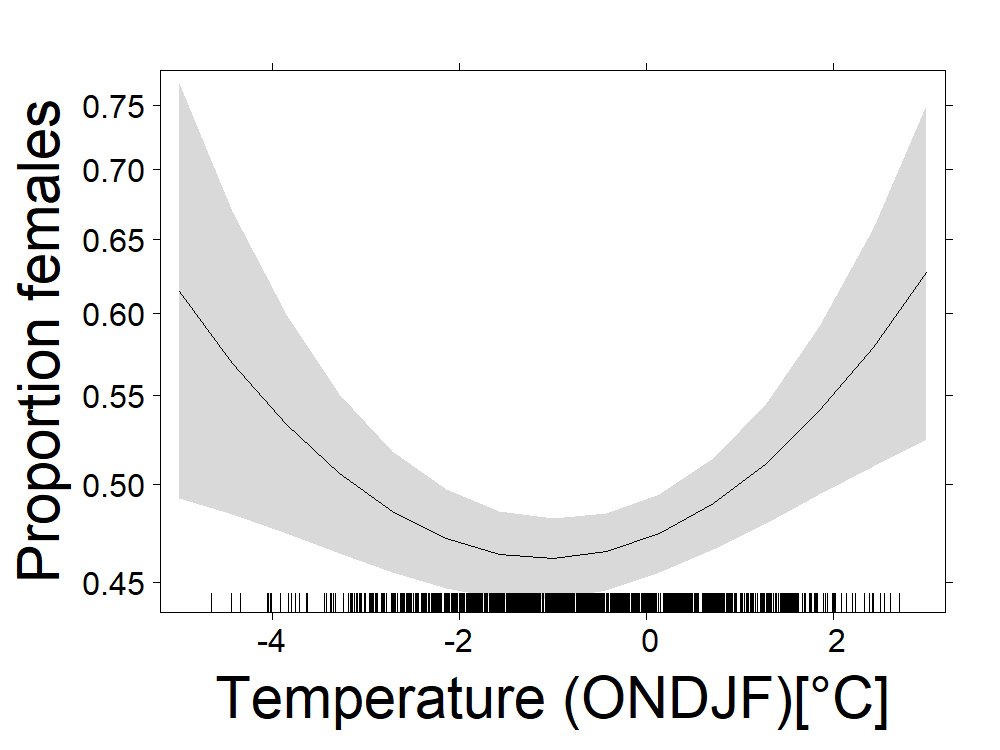

Supplement: Supplementary file 5 — Fig S5 [file ECE3-12-e8938-s009.tiff]

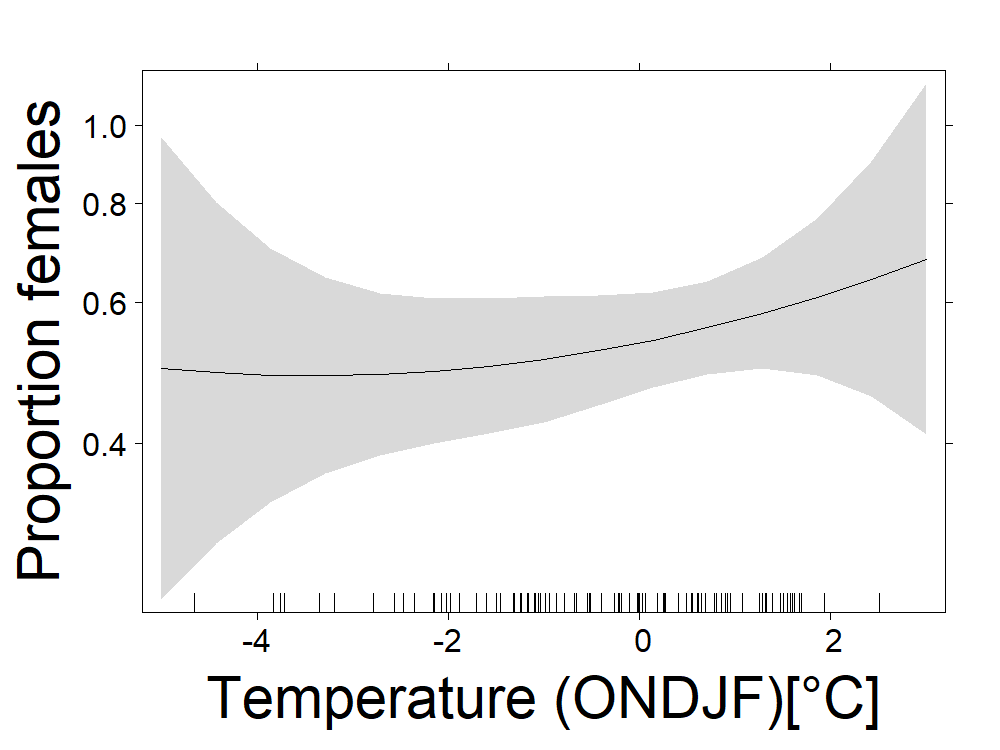

Supplement: Supplementary file 6 — Fig S6 [file ECE3-12-e8938-s005.tiff]

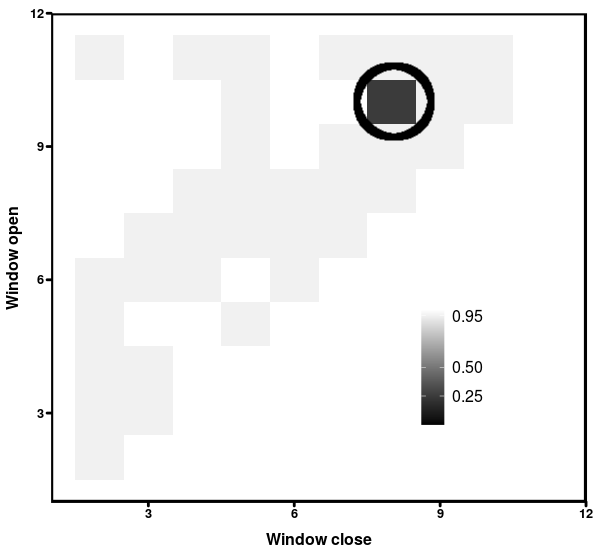

Supplement: Supplementary file 7 — Fig S7 [file ECE3-12-e8938-s006.tif]

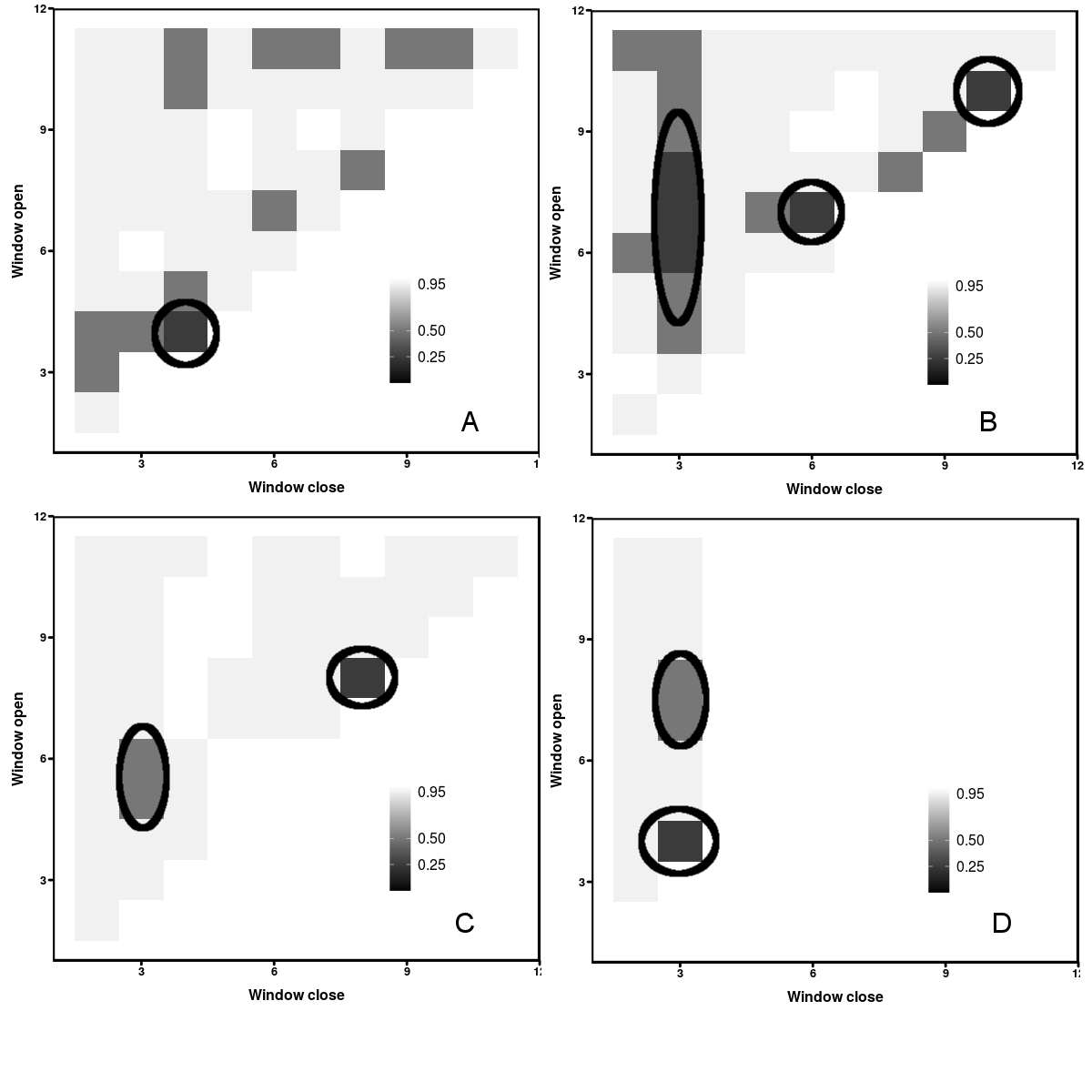

Supplement: Supplementary file 8 — Fig S8 [file ECE3-12-e8938-s008.tif]
