# Supplementary material for: Evidence for a male‐biased sex ratio in the offspring of a large herbivore: The role of environmental conditions in the sex ratio variation
Source: Ecol Evol. 2022 May 19;12(5):e8938. doi: 10.1002/ece3.8938 (PMC9120210; doi:10.1002/ece3.8938)
Supplement: Supplementary file 9 — Table S1‐S3 [file ECE3-12-e8938-s002.docx]

Appendix

Table S1 *References providing information on the number of male and female roe deer offspring, sorted by the year of publication. For references obtained from the reference list of searched publications, the reference of the secondary reference source is provided (Secondary reference source). Information on the author(s), the year of publication (Reference, Author and year), the country (Country) where the study was conducted, the time span (Time) of the study, the number of offspring considered (Sample size), the search engine used (Search engine), the date of the search event (Date), and the key words used in the search is provided (Key words). Each reference obtained an id (Ref.). The references are provided in Table S4. For the case that a reference presented data on distinct populations and/or time periods we included an id for a specific data set (ID-Data set). In case that the data presented were part in a more holistic data collection, we marked the reference by an asterisk, provided the reference containing the broader data set in brackets and used the asterisk for the column “Sample size”. The abbreviation s.o. means “see above”. In case that a study did not provide precise information we used the term NA. As the aim of the systematic literature search was to identify publications providing data on the sex ratio at birth, the list of references presenting data on roe deer offspring older than 2 months is not necessarily complete.*

| ID-Data set | Ref. | Secondary reference source | Reference, Author and year | Country | Time | Sample size | Search engine | Date | | Key words |
| --- | --- | --- | --- | --- | --- | --- | --- | --- | --- | --- |
| 1 | 1 | Ellenberg 1978 | Andersen 1953 | Denmark | 1950 | 91 | - | | - | - |
| 2 | 2 | Ellenberg 1978 | Kurt and Sägesser 1965 | Switzerland | 1965 | 203 | - | | - | - |
| 3 | 3 | Danilkin 1996 | Arabuli 1966 | Georgia | NA | 16 | - | | - | - |
| 4 | 4 | Danilkin 1996 | Nikolandic 1968 | Croatia | 1965–1966 | 186 | - | | - | - |
| 5 | 5 | Ellenberg 1978 | Kurt 1968 | Switzerland | 1963–1964 | 679 | - | | - | - |
| 6 | 6 | Ellenberg 1978 | Prior 1968 | Great Britain | 1968 | 55 | - | | - | - |
| 7 | 7 | Ellenberg 1978 | Espmark 1969 | Sweden | 1957, 1964–1967 | 16 | - | | - | - |
| 8 | 8 | Ellenberg 1978 | Borg 1971 | Sweden | 1948–1969 | 108 | - | | - | - |
| 9 | 8 | Ellenberg 1978 | Borg 1971 | Sweden | 1956–1969 | 475 | - | | - | - |
| 10 | 8 | Ellenberg 1978 | Borg 1971 | Sweden | 1948–1969 | 1100 | - | | - | - |
| 11 | 9 | Ellenberg 1978 | Strandgaard 1972 | Denmark | NA | 168 | - | | - | - |
| 12 | 9 | Ellenberg 1978 | Strandgaard 1972 | Denmark | NA | 97 | - | | - | - |
| 13 | 9 | Ellenberg 1978 | Strandgaard 1972 | Denmark | 1970 | 36 | - | | - | - |
| 14 | 10 | Ellenberg 1978 | Georgii 1973 | Germany | 1972–1973 | 117 | - | | - | - |
| 15 | 11 | Ellenberg 1978 | Wandeler 1975 | Switzerland | 1964–1972 | 262 | - | | - | - |
| 16 | 12 | Danilkin 1996 | Bluzma 1975 | Lithuania | 1964–1974 | 389 | - | | - | - |
| 17 | 13 | Ellenberg 1978 | Blankenhorn 1978 * (Müri 1999) | Switzerland | 1971–1977 | * | - | | - | - |
| 18 | 14 | Müri 1999 | Müri 1978 | Switzerland | 1974–1978 | 39 | - | | - | - |
| 19 | 15 | - | Ellenberg 1978 | Germany | 1974 | 21 | Web of Science | | 30.06.2021 | (Roe deer OR capreolus capreolus) AND sex ratio at birth OR secondary sex ratio OR primary sex ratio |
| 20 | 15 | - | Ellenberg 1978 | Germany | 1972–1976 | 147 | s.o. | | s.o. | s.o. |
| 21 | 15 | - | Ellenberg 1978 | Germany | 1974–1976 | 46 | s.o. | | s.o. | s.o. |
| 22 | 15 | - | Ellenberg 1978 | Germany | 1976 | 65 | s.o. | | s.o. | s.o. |
| 23 | 15 | - | Ellenberg 1978 | Germany | 1972–1974 | 36 | s.o. | | s.o. | s.o. |
| 24 | 15 | - | Ellenberg 1978 | Germany | 1974–1975 | 44 | s.o. | | s.o. | s.o. |
| 25 | 15 | - | Ellenberg 1978 | Netherlands | 1968–1975 | 361 | s.o. | | s.o. | s.o. |
| 26 | 16 | - | Fruzinski and Labudski 1982 | Poland | 1975–1980 | 111 | Google Scholar | | 02.07.2021 | Sex ratio AND Roe deer |
| 27 | 17 | - | Kaluziński 1982 | Poland | 1973–1978 | 298 | Google Scholar | | 02.07.2021 | s.o. |
| 28 | 18 | Flajšman 2017 | Stubbe et al. 1982 | Germany | 1961–1981 | 166 | - | | - | - |
| 29 | 19 | Danilkin 1996 | Engl 1982 | Austria | 1980–1982 | 763 | - | | - | - |
| 30 | 20 | Gaillard et al. 1993 | Pielowski and Bresinsky 1982 | Poland | 1975 | 42 | Google Scholar | | 02.07.2021 | Sex ratio AND Roe deer |
| 31 | 21 | Danilkin 1996 | Pikula et al. 1985 | Czech Republic | 1977–1983 | 500 | - | | - | - |
| 32 | 22 | Danilkin 1996 | Randweer 1989 | Estonia | 1976–1986 | 179 | - | | - | - |
| 33 | 23 | Hewison and Gaillard 1996 | Hewison 1993 | Great Britain | 1981–1990 | 66 | - | | - | - |
| 34 | 23 | s.o. | s.o. | s.o. | 1983–1990 | 28 | - | | - | - |
| 35 | 23 | s.o. | s.o. | s.o. | 1982–1990 | 5 | - | | - | - |
| 36 | 23 | s.o. | s.o. | s.o. | 1983–1990 | 92 | - | | - | - |
| 37 | 23 | s.o. | s.o. | s.o. | 1983–1990 | 85 | - | | - | - |
| 38 | 23 | s.o. | s.o. | s.o. | 1985–1990 | 38 | - | | - | - |
| 39 | 23 | s.o. | s.o. | s.o. | 1982–1990 | 294 | - | | - | - |
| 40 | 23 | s.o. | s.o. | s.o. | 1985–1990 | 33 | - | | - | - |
| 41 | 23 | s.o. | s.o. | s.o. | 1979–1990 | 401 | - | | - | - |
| 42 | 23 | s.o. | s.o. | s.o. | 1986–1990 | 91 | - | | - | - |
| 43 | 23 | s.o. | s.o. | s.o. | 1985–1990 | 53 | - | | - | - |
| 44 | 23 | s.o. | s.o. | s.o. | 1985–1990 | 46 | – | | - | - |
| 45 | 23 | s.o. | s.o. | s.o. | 1970–1990 | 162 | - | | - | - |
| 46 | 23 | s.o. | s.o. | s.o. | 1983–1990 | 145 | - | | - | - |
| 47 | 23 | s.o. | s.o. | s.o. | 1985–1990 | 61 | - | | - | - |
| 48 | 24 | Wauters et al. 1995 | Gaillard et al. 1993 *  (Hewison et al. 1999) | France | 1985–1989 | * | - | | - | - |
| 49 | 25 | Hewison and Gaillard 1996 | Wauters et al. 1995 | Belgium | 1987–1993 | 142 | Web of Science | | 30.06.2021 | (Roe deer OR capreolus capreolus) AND sex ratio at birth OR secondary sex ratio OR primary sex ratio |
| 50 | 26 | Flajšman 2017 | Danilkin 1996 | Russia | NA | 116 | - | | - | - |
| 51 | 27 |  | Hewison and Gaillard 1996* (Hewison 1993) | Great Britain | 1983–1990 | * | Web of Science | | 30.06.2021 | (Roe deer OR capreolus capreolus) AND sex ratio at birth OR secondary sex ratio OR primary sex ratio |
| 52 | 28 |  | Aanes and Andersen 1996 | Norway | 1992–1993 | 45 | Web of Science | | 30.06.2021 | s.o. |
| 53 | 29 | Hewison et al. 1999 | Andersen and Linnell 1996 * (Linnel and Andersen 1998) | Norway |  | * | - | | - | - |
| 54 | 30 | Plard et al. 2014 | Linnell and Andersen 1998 | Norway | 1991–1994 | 293 | - | | - | - |
| 55 | 31 |  | Hewison et al. 1999 *  (Linnell and Andersen 1998 AND Plard et al. 2014) | Norway and France | 1991–1994  and  1985–1996 | * | Web of Science | | 30.06.2021 | (Roe deer OR capreolus capreolus) AND sex ratio at birth OR secondary sex ratio OR primary sex ratio |
| 56 | 32 |  | Müri 1999 | Switzerland | 1971–1995 | 3645 | Web of Science | | 30.06.2021 | s.o. |
| 57 | 33 | Focardi 2002 | Mauget et al. 1999 * (Pettorelli et al. 2003) | France | NA | * | - | | - | - |
| 58 | 34 |  | Quesada and Carranza 2000 | Spain | 1994–1995 | 134 | Google Scholar | | 02.07.2021 | Sex ratio AND Roe deer |
| 59 | 35 |  | Focardi 2002 | Italy | 1995–1999 | 108 | Web of Science | | 30.06.2021 | (Roe deer OR capreolus capreolus) AND sex ratio at birth OR secondary sex ratio OR primary sex ratio |
| 60 | 36 | Mysterud and Østbye 2006 | Pettorelli et al. 2003 | France | 1978–2001 | 1235 | - | | - | - |
| 61 | 37 |  | Pelliccioni et al. 2004 | Italy | 1997–2000 | 78 | Google Scholar | | 02.07.2021 | Sex ratio AND Roe deer |
| 62 | 38 | MacDonald and Johnsen 2008 | Hewison et al. 2005 *  (Plard et al. 2014) | France* and Sweden | NA | NA | - | | - | - |
| 63 | 39 | Authors | Signer and Jenny 2006 | Switzerland | 1972–2005 | 5158 | - | | - | - |
| 64 | 40 |  | Majzinger 2006 | Hungary | 2002–2004 | 53 | Google Scholar | | 02.07.2021 | Sex ratio AND Roe deer |
| 65 | 40 |  | Majzinger 2006 | Hungary | 2002–2004 | 20 | Google Scholar | | 02.07.2021 | s.o. |
| 66 | 41 |  | Mysterud and Østbye 2006 | Norway | 1985–2001 | 109 | Google Scholar | | 02.07.2021 | s.o. |
| 67 | 42 |  | MacDonald and Johnsen 2008 | Great Britain | 1994–2005 | 69 | Web of Science | | 30.06.2021 | (Roe deer OR capreolus capreolus) AND sex ratio at birth OR secondary sex ratio OR primary sex ratio |
| 68 | 42 |  | s.o. | s.o. | 1994–2005 | 90 | s.o. | | s.o. | s.o. |
| 69 | 42 |  | s.o. | s.o. | 1994–2005 | 91 | s.o. | | s.o. | s.o. |
| 70 | 42 |  | s.o. | s.o. | 1994–2005 | 116 | s.o. | | s.o. | s.o. |
| 71 | 42 |  | s.o. | s.o. | 1994–2005 | 49 | s.o. | | s.o. | s.o. |
| 72 | 42 |  | s.o. | s.o. | 1994–2005 | 54 | s.o. | | s.o. | s.o. |
| 73 | 42 |  | s.o. | s.o. | 1994–2005 | 52 | s.o. | | s.o. | s.o. |
| 74 | 42 |  | s.o. | s.o. | 1994–2005 | 76 | s.o. | | s.o. | s.o. |
| 75 | 42 |  | s.o. | s.o. | 1994–2005 | 51 | s.o. | | s.o. | s.o. |
| 76 | 42 |  | s.o. | s.o. | 1994–2005 | 54 | s.o. | | s.o. | s.o. |
| 77 | 42 |  | s.o. | s.o. | 1994–2005 | 54 | s.o. | | s.o. | s.o. |
| 78 | 42 |  | s.o. | s.o. | 1994–2005 | 240 | s.o. | | s.o. | s.o. |
| 79 | 42 |  | s.o. | s.o. | 1994–2005 | 68 | s.o. | | s.o. | s.o. |
| 80 | 42 |  | s.o. | s.o. | 1994–2005 | 62 | s.o. | | s.o. | s.o. |
| 81 | 42 |  | s.o. | s.o. | 1994–2005 | 304 | s.o. | | s.o. | s.o. |
| 82 | 42 |  | s.o. | s.o. | 1994–2005 | 188 | s.o. | | s.o. | s.o. |
| 83 | 42 |  | s.o. | s.o. | 1994–2005 | 664 | s.o. | | s.o. | s.o. |
| 84 | 42 |  | s.o. | s.o. | 1994–2005 | 108 | s.o. | | s.o. | s.o. |
| 85 | 42 |  | s.o. | s.o. | 1994–2005 | 272 | s.o. | | s.o. | s.o. |
| 86 | 43 | Plard 2014 | Plard et al. 2013 * (Plard et al. 2014) | France | 1985–2010 | * | - | | - | - |
| 87 | 44 |  | Plard et al. 2014 | France | 1985–2011 | 1083 | Google Scholar | | 02.07.2021 | Sex ratio AND Roe deer |
| 88 | 45 | Authors | Flajšman 2017 | Slovenia | 2014–2017 | 39 | - | | - | - |
| 89 | 46 | Authors | Flajšman 2017 | Slovenia | 2013–2015 | 360 | - | | - | - |
| 90 | 47 |  | This publication 2022 | Germany (Baden-Württemberg) | 1972–2019 | 12473 | NA | | - | - |
| 91 | 48 |  | This publication 2022 | Germany (Niederfinow) | 2004–2019 | 223 | NA | | - | - |

*Table S2 Proportion of female offspring for each study site* *with information on the prenatal sex ratio of roe deer offspring (calculated using data with two decimal places), its 95% confidence interval (CI_95_), and information on the sample size, location, coordinates (in case of several distinct locations, a representative coordinate was chosen) and living conditions. Bold entries refer to deviations from parity.*

| ID (see Table S1 for further information) | Reference | Proportion of female offspring | CI_95_ | Sample Size | Location | Coordinate in WGS 84 (Lat, Long) |
| --- | --- | --- | --- | --- | --- | --- |
| 6 | 6 | 0.47 | [0.34, 0.61] | 55 | Cranborne Chase  (Great Britain) | 50.97, -2.07 |
| 8 | 8 | 0.56 | [0.46, 0.65] | 108 | Diverse  (Sweden) | 62.39, 16.32 |
| 14 | 10 | 0.57 | [0.48,0.66] | 117 | Bavaria  (Germany) | 49.23, 11.77 |
| **15** | **11** | **0.45** | **[0.36, 0.49]** | **262** | **Diverse (**Switzerland**)** | **47.06, 7.58** |
| 26 | 16 | 0.56 | [0.46, 0.65] | 111 | Zielonka  (Poland) | 52.39, 17.21 |
| 27 | 17 | 0.46 | [0.4, 0.52] | 298 | Czempiïn  (Poland) | 52.15, 16.77 |
| 28 | 18 | 0.48 | [0.4, 0.55] | 166 | Hakel  (Germany) | 51.89, 11.33 |
| 33 | 23 | 0.39 | [0.28,0.52] | 66 | Craigellachie  (Great Britain) | 57.52, -3.17 |
| 36 | 23 | 0.42 | [0.32,0.53] | 92 | Spadeadam  (Great Britain) | 55.02, -2.64 |
| 37 | 23 | 0.39 | [0.28,0.5] | 85 | Kershope  (Great Britain) | 55.14, -2.74 |
| 39 | 23 | 0.5 | [0.44,0.56] | 294 | Pickering  (Great Britain) | 54.37, -1.24 |
| 41 | 23 | 0.47 | [0.42,0.52] | 401 | Thetford  (Great Britain) | 52.43, 0.69 |
| 42 | 23 | 0.48 | [0.38,0.59] | 91 | Salisbury Plain  (Great Britain) | 51.21, -2.03 |
| 43 | 23 | 0.43 | [0.3, 0.58] | 53 | Bramley  (Great Britain) | 51.33, -1.11 |
| 45 | 23 | 0.51 | [0.43,0.59] | 162 | Alice Holt  (Great Britain) | 51.18, -0.85 |
| 46 | 23 | 0.46 | [0.38,0.55] | 145 | Ringwood  (Great Britain) | 50.85, -1.81 |
| 47 | 23 | 0.46 | [0.33,0.59] | 61 | Lulworth  (Great Britain) | 50.62, -2.24 |
| 48 | 24 | 0.49 | [0.41, 0.58] | 142 | Limburg  (Belgium) | 50.65, 5.93 |
| 64 | 40 | 0.53 | [0.39,0.67] | 53 | Great Hungarian Plain (Hungary) | 47.65, 21.08 |
| 67 | 42 | 0.38 | [0.26, 0.5] | 69 | Aldershot  (Great Britain) | 51.27, -0.76 |
| 68 | 42 | 0.52 | [0.41, 0.63] | 90 | Ash  (Great Britain) | 51.27, -0.7 |
| 69 | 42 | 0.51 | [0.4, 0.61] | 91 | Bordon/Longmark  (Great Britain) | 51.09, -0.92 |
| 70 | 42 | 0.41 | [0.32, 0.51] | 116 | Bramley  (Great Britain) | 51.33, -1.11 |
| 72 | 42 | 0.44 | [0.31, 0.59] | 54 | Glendouglas  (Great Britain) | 56.14, -4.72 |
| 73 | 42 | 0.58 | [0.43, 0.71] | 52 | Hawley/Minley  (Great Britain) | 51.31, -0.82 |
| 74 | 42 | 0.47 | [0.36,0.59] | 76 | Kirkcudbright  (Great Britain) | 54.83, -4.04 |
| 75 | 42 | 0.47 | [0.33, 0.62] | 51 | Lulworth  (Great Britain) | 50.62, -2.24 |
| 76 | 42 | 0.56 | [0.41, 0.69] | 54 | Otterburn  (Great Britain) | 55.24, -2.17 |
| 77 | 42 | 0.43 | [0.29, 0.57] | 54 | Pirbright  (Great Britain) | 51.29, -0.65 |
| 78 | 42 | 0.54 | [0.48, 0.61] | 240 | Porton  (Great Britain) | 51.12, -1.72 |
| 79 | 42 | 0.49 | [0.36, 0.61] | 68 | Sandhurst  (Great Britain) | 51.36, -0.79 |
| 80 | 42 | 0.45 | [0.32, 0.58] | 62 | Spadeadam  (Great Britain) | 55.02, -2.65 |
| **81** | **42** | **0.44** | **[0.38, 0.5[** | **304** | **SPTAC**  (Great Britain) | **51.04, -1.8** |
| 82 | 42 | 0.49 | [0.42, 0.56] | 188 | SPTAE  (Great Britain) | 51.04, -1.8 |
| **83** | **42** | **0.44** | **[0.41, 0.48]** | **664** | **SPTAW**  (Great Britain) | **51.04, -1.8** |
| 84 | 42 | 0.55 | [0.45, 0.64] | 108 | Stanford  (Great Britain) | 52.78, 0.74 |

*Table S3* *Results of Eq. 1 using the sex of roe deer offspring as the response variable and the parameters used by Müri (1999). Monthly mean precipitation in May, June, January, February, and the monthly mean temperature for May and June.*

Variable Estimate Standard error p-value

Intercept -8.154e-01 1.169e-01 -

Precipitation [Jan] 1.250e-04 3.270e-04 0.702

Precipitation [Feb] 1.932e-04 3.234e-04 0.550

Temperature [Jun (t-1)] 7.072e-03 7.615e-03 0.353

Temperature [May (t-1)] -1.284e-03 7.133e-03 0.857

Precipitation [May (t-1)] 1.399e-05 2.874e-04 0.961

Precipitation [Jun (t-1)] -1.045e-04 3.868e-04 0.787
