# Supplementary material for: Evidence for a male‐biased sex ratio in the offspring of a large herbivore: The role of environmental conditions in the sex ratio variation
Source: Ecol Evol. 2022 May 19;12(5):e8938. doi: 10.1002/ece3.8938 (PMC9120210; doi:10.1002/ece3.8938)
Supplement: Supplementary file 10 — Table S4 [file ECE3-12-e8938-s001.docx]

*Table S4 References cited in Table S1*

| Aanes, R. & Andersen, R. (1996) The effects of sex, time of birth, and habitat on the vulnerability of roe deer fawns to red fox predation. *Canadian Journal of Zoology*, **74**, 1857–1865.  Andersen, J. (1953) Analysis of a Danish roe-deer population (Capreolus capreolus (L.)) based upon the extermination of the total stock. *Danish Review of Game Biology*, **2**, 127–155.  Andersen, R. & Linnell, J.D.C. (1996) Variation in maternal investment in a small cervid; the effects of cohort, sex, litter size and time of birth in roe deer (Capreolus capreolus) fawns. *Oecologia*, **109**, 74–79.  Arabuli. (1966) Roe deer in East Georgia. *Author’s abstract of the Dissert. for the degree of Cand. Sc. (Biology)*, 22.  Blankenhorn, H.J. (1978) Die Kitzmarkierungsaktion 1971-1977. *Schweizerjäger*, **6**, 362–367.  Bluzma, P.P. (1975) *The Ecology and Morphology of Lithuanian Roe Deer*. Moscow.  Borg, K. (1971) *On Mortality and Reproduction of Roe Deer in Sweden during the Period 1948-1969*.  Danilkin, A. (1996) Population structure and dynamics. *Behavioural ecology of Sibirian and European roe deer*, pp. 200–219.  Ellenberg, H. (1978) Zur Populationsökologie des Rehes (Capreolus capreolus L., Cervidae) in Mitteleuropa. *Spixiana*, **2**, 1–212.  Engl, D. (1982) Rehwildmarkierung. *Österreichs Weidwerk*, 210–211.  Espmark, Y. (1969) Mother-young relations and development of behaviour in roe deer. *Viltrevy*, **6**, 461–540.  Flajšman, K. (2017) *Effects of Individual, Population and Environmental Factors on Reproductive Success of Roe Deer (Capreolus Capreolus L .)*. Ljubljana.  Focardi, S., Pelliccioni, E., Petrucco, R. & Toso, S. (2002) Spatial patterns and density dependence in the dynamics of a roe deer (Capreolus capreolus) population in central Italy. *Oecologia*, **130**, 411–419.  Fruziński, B. & Łabudzki, L. (1982) Demographic processes in a forest roe deer population. *Acta Theriologica*, **27**, 365–375.  Gaillard, J.M., Delorme, D., Jullien, J.M. & Tatin, D. (1993) Timing and synchrony of births in roe deer. *Journal of Mammology*, **74**, 738–744.  Georgii, B. (1973) Nebennierengewichte und Corpora Lutea bei Rehwild. *XIth International Congress of Game Biologists*, pp. 125–131.  Hewison, M.A.J. (1993) *The Reproductive Performance of Roe Deer in Relation to Environmental and Genetic Factors*. Southampton.  Hewison, A.J.M., Andersen, R., Gaillard, J.M., Linnell, J.D.C. & Delorme, D. (1999) Contradictory findings in studies of sex ratio variation in roe deer (Capreolus capreolus). *Behavioral Ecology and Sociobiology*, **45**, 339–348.  Hewison, A.J.M. & Gaillard, J.M. (1996) Birth-sex ratios and local resource competition in roe deer, Capreolus capreolus. *Behavioral Ecology*, **7**, 461–464.  Hewison, A.J.M., Gaillard, J.M., Kjellander, P., Toïgo, C., Liberg, O. & Delorme, D. (2005) Big mothers invest more in daughters - Reversed sex allocation in a weakly polygynous mammal. *Ecology Letters*, **8**, 430–437.  Kałuziński, J. (1982) Dynamics and structure of a field roe deer population. *Acta Theriologica*, **27**, 385–408.  Kurt, F. (1968) Zusammenhänge zwischen Verhalten und Fortpflanzungsleistung beim Reh (Capreolus capreolus L.). *Zeitschrift für Jagdwissenschaft*, **14**, 97–106.  Linnell, J.D.C. & Andersen, R. (1998) Timing and synchrony of birth in a hider species, the roe deer Capreolus capreolus. *Journal of Zoology*, **244**, 497–504.  MacDonald, D.W. & Johnson, P.J. (2008) Sex ratio variation and mixed pairs in roe deer: Evidence for control of sex allocation? *Oecologia*, **158**, 361–370.  Majzinger, I. (2006) Comparison of Reproductive Performance of the Roe Deer (Capreolus capreolus, L.) Among Different Regions. *Acta Agraria Debreceniensis*, **78**, 41–46.  Mateos-Quesada, P. & Carranza, J. (2000) Reproductive patterns of roe deer in Central Spain. *Etologia*, **8**, 17–20.  Mauget, C., Mauget, R. & Sempéré, A. (1999) Energy expenditure in European roe deer fawns during the suckling period and its relationship with maternal reproductive cost. *Canadian Journal of Zoology*, **77**, 389–396.  Müri, H. (1978) Beobachtungen zu Setzzeit und Geschlechterverhältnis bei Gehege-Rehen. *Schweizer Jäger*, **63**, 465–466.  Müri, H. (1999) Witterung, Fortspflanzungsgeschehen und Bestandesdichte beim Reh. *Zeitschrift für Jagdwissenschaft*, **45**, 88–95.  Mysterud, A. & Østbye, E. (2006) Effect of climate and density on individual and population growth of roe deer Capreolus capreolus at northern latitudes: the Lier valley, Norway. *Wildlife Biology*, **12**, 321–329.  Nikolandic, D. (1968) Ökologische Charakteristik der Rehpopulation im Distrikt Belje, 73–95.  Pelliccioni, E.R., Scremin, M. & Toso, S. (2004) Early body development of roe deer Capreolus capreolus in a sub-Mediterranean ecosystem. *Wildlife Biology*, **10**, 107–113.  Pettorelli, N., Dray, S., Gaillard, J.M., Chessel, D., Duncan, P., Illius, A., Guillon, N., Klein, F. & Van Laere, G. (2003) Spatial variation in springtime food resources influences the winter body mass of roe deer fawns. *Oecologia*, **137**, 363–369.  Pielowski, Z. & Bresiński, W. (1982) Population characteristics of roe deer inhabiting a small forest. *Acta Theriologica*, **27**, 409–425.  Pikula, J., Koubek, P., Kratochvil, Z. & Kux, Z. (1985) Age composition of roe deer population in Czechoslovakia. *Acta Sc Nat Brno*, **19**, 1–46.  Plard, F., Gaillard, J.M., Bonenfant, C., Hewison, A.J.M., Delorme, D., Cargnelutti, B., Kjellander, P., Nilsen, E.B. & Coulson, T. (2013) Parturition date for a given female is highly repeatable within five roe deer populations. *Biology letters*, **9:20120841**.  Plard, F., Gaillard, J.M., Coulson, T., Hewison, A.J.M., Delorme, D., Warnant, C. & Bonenfant, C. (2014) Mismatch Between Birth Date and Vegetation Phenology Slows the Demography of Roe Deer ed G.M. Mace. *PLoS Biology*, **12**, e1001828.  Prior, R. (1968) *The Roe Deer of Cranborne Chase. An Ecological Survey*. Oxford University Press.  Randweer, T.E. (1989) *Ecological Peculiarities and Exploitation of the Estonian Roe Deer (Capreolus Capreolus L.) Population*. Moscow.  Sägesser, H. & Kurt, F. (1965) Über die Setzzeit 1965 beim Reh ( Caproelus c . capreolus [ L .]). *Mitteilungen der Naturforschenden Gesellschaft in Bern*, **23**.  Signer, C. & Jenny, H. (2006) Rehkitzmarkierung im Kanton Graubünden 1972-2005, 1–86.  Strandgaard, H. (1972) The Roe Deer (Capreolus capreolus) Population at Kalø and the Factors Regulating its Size. *Danish Review of Game Biology*, **7**, 205.  Stubbe, C., Stubbe, M. & Stubbe, I. (1982) Zur Reproduktion der Rehwildpopulation-Capreolus c. capreolus (L.,1758) -des Wildforschungsgebietes Hakel. *Hercynia*, **19**, 97–109.  Wandeler, A.L. (1975) Die Fortpflanzungsleistung beim Reh (Capreolus capreolus L.) im Berner Mittelland. *Jahrbuch des Naturhistorischen Museums Bern*, pp. 245–301.  Wauters, L.A., de Crombrugghe, S.A., Nour, N. & Matthysen, E. (1995) Do female roe deer in good condition produce more sons than daughters. *Behavioral Ecology and Sociobiology*, **37**, 189–193. |
| --- |
